# Address

**Published:** 1872-04

**Authors:** James Taylor


### ADDRESS

Read before the Graduating Class of the Ohio Dental College, at its Twenty-Sixth Commencement.

BY. DR. JAS. TAYLOR.

GENTLEMEN OF THE GRADUATING CLASS: Custom imposes on me the pleasant duty of offering a few remarks on this occasion. You must not expect, however, a lecture on any subject of which these diplomas bear evidence of attainment. Yet we would caution you now, not to think that even those studies, on which you have borne so creditable an examination, should be laid aside as finished. You are now, we take it, only the more fully prepared to prosecute these studies with that comprehension and accuracy which they deserve. We are all learners, and this is your commencement. When we reflect how little of our knowledge is perfect, how little is stamped with that certainty which renders no more light or investigation necessary; we must see and feel that there is no stopping place to loiter in.

Revelations of scientific research is constantly unfolding new views of almost every principle and practice of our profession. These new views often so attract and excite our imagination, that at first we are not prepared to soberly and carefully adjust their relative bearing on our science. Take all our improvements which have been made in the last thirty years, and you can carefully note step by step the development, and often you see that many minds and many hands have helped to work out the result. Often he who has put on the finishing touch and claims all the credit, has, indeed, done less than many others. The first step in a difficult problem is often the most arduous, and contains the germ of a creative genius.

We can not say that any operation you may be called upon to perform is perfect in all its detail of execution.

We can say, however, with perfect assurance, that ten, twenty, or even thirty, years of practice will leave you much to learn. You must not be surprised if you find out after ten years experiment that certain proposed modes of practice are utter failures. The fact is, we are in a developmental period, and must expect changes in the method of doing things.

You can not feel as fixed in the practice of mechanical dentistry to-day as we felt ten years ago. In this department there is not now (we mean as generally practiced) half the skill and true art brought into requisition as then. The tendency of mechanic art in this progressive age is to machinery, and our profession, we fear, in this specialty of it, has yielded much to the spirit of the age. Was it possible we should soon see whole sets of teeth supplied from our depots like any other piece of merchandise? We wish you not to infer from this that mechanical dentistry fails now to meet the general wants of the public as then—in a certain sense this is not so—but while this want may be as fully supplied, it has been at the expense of true excellence. In seeking to supply the demand through a cheap material, the operation has been reduced in quality as well as price. The photographer occupies much the same position with reference to the noble art of painting, as the worker of rubber does to that of a true dentist. They both supply substitutes which are more in demand for their cheapness than that they approach that which they represent in quality or excellence. A true regard for the best interest of society and our profession with time which tests these things, will, we hope, correct these evils. Have faith in your profession and work for its exaltation.

Some thirty years since, a gentleman then engaged in the practice of dentistry, remarked to me that "he should quit the practice," and assigned as a reason that the study and practice was too circumscribed for an intellectual mind. We told him that he certainly failed to comprehend the full scope of dental science. The fact is, he had scarcely got within the vestibule, and was already eager and impatient for the compensation which alone should reward true merit and praiseworthy effort. Standing, gentlemen, thirty years in advance of this intellectual giant, with a profession so expanded that it is being divided into specialties, you may form some conception of the change which has been wrought. You must recollect that forty years since, three months pupilage and no collegiate course was not very apt to produce an exalted opinion of any profession so easily acquired. A long life of study with the greatest of intellect often results in the conviction that but little in comparison has been learned.

It was this profound ignorance—which, we may say, "abounded"—that first suggested the idea of our dental colleges. We had even then a few here and there who practiced the profession as a specialty of general surgery. They saw that this intolerable ignorance must be broken up or the profession abandoned. Efforts were made to engraft a department of dentistry to some of our medical schools, but failing in this, Prof. C. A. Harris, of Baltimore, Dr. Hayden, of the same city, and two or three practicing physicians organized the Baltimore College of Dental Surgery. This was in 1840—at least the first course of lectures commenced in the fall of that year.

That you may form some conception of what you and the profession owe these gentlemen, and particularly Dr. Harris, who was evidently the prime mover in the matter, we will try and give you an idea of the status of dental surgery at that time.

We go back to 1815, and we find the now justly celebrated and, we believe, the retired veteran of the profession, Dr. Eliazer Parmly, of New York, a young man of eighteen, studying with his brother, carving and manufacturing teeth from the sea-horse. In this year the first gold plug he saw was in a front tooth which had been stopped with gold by

Mr. Waite, of London. This appeared to be a gem in its way, and fully aroused that spirit of emulation which never slept, and still burns brightly in the hale old gentleman.

In a tour shortly after this through the West the Doctor saw, while at Lexington, Ky., a few fillings put in by Dr. Hudson, of Philadelphia, resembling the one of Dr. Waite. He then got a silver plater to roll and hammer down some gold coin as thin as possible, and with this commenced his first gold fillings. During this trip, while at New Orleans, he saw for the first time a porcelain tooth and swaged gold plate. Soon after this, he remarks, "I embarked for London with as full and as strong a determination as I think was ever felt by any human being, never again to touch the shores of my native country until I should know exactly how all these things were done."

From London he goes to Paris, and, fortunately, got in with Maury and completed his mechanical studies with him. Then back to London, and with his brother commenced the practice. He appears all this time to have been haunted by the fine gold plug of Mr. Waite. He calls on him in London. We now give his own words: "But as he (Mr. Waite) was absent, his brother handed me a part of a leaf of gold, the size of which originally must have equaled that of our largest sheets of tin foil, and as thick as one of our common-sized sheets of gold would be of twenty or twenty-five grains. After feeling of the gold I remarked that it seemed soft, yet was very thick, to which he replied: 'Yes, it requires great strength to force it into a tooth, but when firmly pressed in it makes a very solid stopping.' I inquired where it could be had. 'He said he did not know, but believed his brother got it somewhere about St. James.' Most eagerly and faithfully did I look and inquire in vain for it in all the streets, alleys and courts of St. James, but I have never to this day seen any like it."

This gives you some idea, gentlemen, of the difficulties that then surrounded the search for dental knowledge. There was a little light, the glimmer of which aroused the ambition of such a man as Parmly, but O! how many either never caught a ray of this light or lacked the energy to use and improve it.

You must recollect that this talk of Dr. Parmly was in 1842, and you may understand that heavy foil dates back to 1815, and that which was made by the silver plater at Lexington must have been the heaviest of the heavy.

In looking at this hasty sketch of the pupilage of one of our most celebrated dentists, we can not but see the necessity for dental colleges.

Let us come, however, to 1840, and hear what Prof. Harris says in that introductory address of his which inaugurated the first institution of the kind ever known.

He remarks: "Accessible as has been the calling of the dentist to all that were disposed to enter it, and that too without regard to qualification, it has been resorted to by the ignorant and illiterate, and I am sorry to say, in too many instances, by unprincipled individuals, until it now numbers in the United States about twelve hundred, and of which, I think it may be safely asserted not more than one in six possess any just claim to a correct or thorough knowledge of the pursuit.'

We think none of our old practitioners will doubt the truth of this statement. We could for an hour state fact after fact confirmatory of this.

With this state of things the Baltimore school was established, and four years thereafter the Ohio College of Dental Surgery was instituted. Professors Harris and Hayden lived to see a great work accomplished, and particularly the former. His most sanguine expectations were more than realized. By faith his prophetic eye beheld the splendid results to be attained, and this hid from view the long, arduous toil for its accomplishment.

How very few of these pioneers remain to see the result of their labors. Harris is gone. Hayden is gone, and of our own school Professors Cook and Leslie are gone; and of our medical coadjutors Professors Slack, Shotwell, Potter, and Smith are gone.

In all this labor for the advancement of dental science let us not forget our obligations to the medical profession. We look with pride on our professional zeal—the *esprit du corps* which excites so much commendable emulation. We would extend this to all of our brethren of our good old noble

Mother, never forgetting the cordial, fraternal feeling which has been extended to us in every step of our advancement.

Where would the Baltimore School have been without their aid. Half their professors were from the medical profession. Look at our own school. How much are we indebted to the accomplished Shotwell, who for years filled so creditably the chair of anatomy in the Medical College of Ohio, and came to our aid in the same department—then also came to our aid Dr. Potter, a devoted and efficient teacher of anatomy. How many of our graduates cherish the memory of Prof. J. B. Smith, who so long and ably filled the chair of pathology in this institution. His untiring zeal, his devotion to the advancement of the class, his kindness of heart and gentlemanly bearing, endeared him to the students as well as his colleagues.

We believe that Dr. Slack filled the first chair of dental chemistry ever instituted. He was our first professor. A devoted chemist, he honored his profession. He delighted in the study, and taught it with a zeal and enthusiasm unbounded. Not only caries of the teeth, but all diseases of the body were of chemical origin, and through chemical science all should be treated. It was with him the foundation science, and a dental school without a chemical chair should not be thought of.

He was Professor of Chemistry in the old Cincinnati College when cows ran at large on Walnut street, and it is said that one morning he found one of these animals in his laboratory up stairs. How she got there the boys only knew. The Professor labored to drive her down, but at the top of the stairs she would always wheel around and refuse to go. At length he called in the aid of some of the boys. They caught her by the tail and pulled and tugged, but still she would not come. At last the Doctor told the boys to hold on and he would apply the battery. So he charged it to the full and touched the pole to the nose of the cow, and cow and boys were landed on the pavement.

Dr. Cook was the first Professor of Anatomy in this school, and labored with untiring industry to place this chair on a firm and solid basis. Eminent as a dental practitioner, he fully realized the importance of a thorough knowledge of anatomy as the basis of correct dental practice. As a teacher Dr. Cook possessed many admirable traits. Possessing an exalted estimate of the importance of dental science, he ever sought to impress this not only on the student but the public at large. His removal from our city deprived us of his valuable labors, and he ultimately resumed the practice of medicine in an adjoining county, where he spent several years in extensive practice. Until death he looked with special interest to the success of this institution.

Dr. Leslie occupied the chair of Mechanical Dentistry and Metallurgy for one session in this school. He was one of our early graduates, and on more than one occasion showed his attachment to his Alma Mater. His early life and labor had eminently fitted him for the chair to which he was assigned. We are satisfied, had his professional and other duties permitted him to have filled the chair for a few years, he would have worked it up to a degree of excellence which would have been felt and appreciated by the profession at large.

His practical mind took in at once all the minute details of his chair, and his thorough mastery of everything he undertook enabled him clearly and forcibly to impress these things on the minds of the students.

We shall ever recollect with great satisfaction his zeal and efficient aid in our effort to liquidate the debt which pressed like an incubus on this institution. He then resided in St. Louis, whither we journeyed to test the liberality of our brethren of that city. Dr. Leslie at once entered into our plans, called the profession together, took a share of stock himself, and went with me from office to office until we raised fifteen hundred dollars on our debt.

We came away saying God bless the brethren of St. Louis, and make all our graduates like Dr. Leslie. An institution thus aided, cherished and founded by a band of the most noble men that ever adorned any profession should never fail.

But, gentlemen, while so many have passed from our midst and have left such cherished memories and such noble records of usefulness, there are still a few of our early co-laborers left to give us their countenance and support, and who feel as strong an interest in the success of this institution as ever

Let us bring to remembrance those honored ones, and wish them many years of continued usefulness.

Dr. M. Rogers filled the chair of Pathology and Therapeutics for two years. For many years he held a prominent position in this city as a dental operator. Faithful and laborious in his profession, his medical studies had prepared him especially for the chair to which he was assigned. Being one of the originators of this school, and having retired from the profession. this passing notice, although living, we hope is not out of place.

Prof. Thos. Wood, M. D., will ever be honored for his truly able and interesting anatomical teaching in this school. His two shares of stock speak well for the interest he has always felt in the cause of dental science.

There is another whose long and faithful labors have endeared him to us all, and who not only as teacher but stockholder helped to put upon a firm basis this institution of your Alma Mater. Throughout the country you will find his name on medical as well as dental diplomas—this is Professor G. Mendenhall, M. D.

We name also C. B. Chapman, M. D., Prof. of Chemistry, a devoted and most excellent teacher. Then Dr. Allen, now of New York. Ever ready to teach and be taught, his name brings up continuous gum work and will be remembered as long as artificial dentures are worn.

We also have Drs. Jones, Van Emon, and Richardson, whose admirable work on Mechanical Dentistry you have all seen. But we forbear, gentlemen. It is needless to say that the celebrated Watt family as well as the ubiquitous Smith have been represented here.

We look with just pride on the rapid advancement of our noble science. The twelve hundred have grown to twelve thousand or more, and instead of one in six only being worthy of recognition, let us hope that five out of every six are worthy of our confidence. Every city and town of any size turns out now gold fillings which compare not unfavorably with the beautiful gem of Mr. Waite.

In conclusion, we would impress on your minds one thought, which is this—that dental science is medical science. The schools which inaugurated special dental instruction were almost exclusively inaugurated by medical men—even those who were practicing the specialty of dentistry were regular medical practitioners.

Your curriculum of study is almost the same—adding on full as much as you lop off. Those diplomas confer the degree of doctor. It is a time-honored title, and all those who are entitled to it now greet you as brethren.

Let your emulation be to rank yourselves in the front, and help on to greater victories over disease.

It is a time-honored custom in this institution to present each graduate with a copy of the Holy Scriptures—the book of books. Its Author being all wise, you can take its teaching with implicit confidence. It has withstood for eighteen centuries the assaults of infidel skepticism, but to-day its truths have a deeper hold on the throbbing heart of the world than ever before. It proclaims and makes known to you that infinite wisdom and almighty power which is so manifest in this mysterious organization of ours. The dental organs alone in their development unfold a page of recorded wisdom and forethought which infidelity can never refute.

This book has introduced a Christian civilization which has done more for art and science than all other books put together. Outside of this civilization your profession is scarcely known. Make this book, which is God's word to us all, your daily study. Conform your lives to its precepts, and your greatest good is sure to be attained.

